# Backward spoof surface wave in plasmonic metamaterial of ultrathin metallic structure

**DOI:** 10.1038/srep20448

**Published:** 2016-02-04

**Authors:** Xiaoyong Liu, Yijun Feng, Bo Zhu, Junming Zhao, Tian Jiang

**Affiliations:** 1 Department of Electronic Engineering, School of Electronic Science and Engineering, Nanjing University, Nanjing, 210093, China

## Abstract

Backward wave with anti-parallel phase and group velocities is one of the basic properties associated with negative refraction and sub-diffraction image that have attracted considerable interest in the context of photonic metamaterials. It has been predicted theoretically that some plasmonic structures can also support backward wave propagation of surface plasmon polaritons (SPPs), however direct experimental demonstration has not been reported, to the best of our knowledge. In this paper, a specially designed plasmonic metamaterial of corrugated metallic strip has been proposed that can support backward spoof SPP wave propagation. The dispersion analysis, the full electromagnetic field simulation and the transmission measurement of the plasmonic metamaterial waveguide have clearly validated the backward wave propagation with dispersion relation possessing negative slope and opposite directions of group and phase velocities. As a further verification and application, a contra-directional coupler is designed and tested that can route the microwave signal to opposite terminals at different operating frequencies, indicating new application opportunities of plasmonic metamaterial in integrated functional devices and circuits for microwave and terahertz radiation.

Backward electromagnetic (EM) wave is usually defined as the wave propagating with opposite directions of its phase and group velocities^1^. It has attracted considerable attention in the past decade in the context of metamaterials, especially the left-handed or double negative metamaterials with simultaneously negative permittivity and permeability^2^,^3^. Backward wave is one of the important peculiar property associated with metamaterials that forms the basis for negative refraction^3^ and underlies other interesting phenomena, such as the perfect lenses^4^,^5^, or hyperlenses^6^,^7^ that aim at achieving optical resolutions overcoming the diffraction limit with obvious potential for optical imaging and lithography. Backward wave has also been well demonstrated in the circuit analogy of left-handed metamaterials — the left-handed transmission lines^8^,^9^, and been utilized in many novel EM components or antenna concepts^10^.

Surface plasmon polaritons (SPPs) are surface EM waves propagating along the dielectric-metal interface at optical frequencies^11^,^12^. Owing to their strong field confinement to sub-wavelength scale and consequent field enhancement at the dielectric-metal interface, SPPs could lead to the overcome of the diffraction limit^13^, and miniaturized optical integrated circuits and devices with scales smaller than the light wavelength^14^,^15^,^16^. For the SPP wave on a dielectric-metal interface, the EM wave is forward inside the dielectric but backward inside the metal having a negative permittivity^17^. However, large fraction of the energy flows in the dielectric making the net behavior of the SPP wave a forward wave. Many theoretical studies have been made to explore the backward wave propagation of SPPs, which reveal that properly designed tri-layer waveguide, either a metal-insulator-metal (MIM) or an insulator-metal-insulator (IMI) structure, may support the backward propagation or negative index mode^18^,^22^. Although interesting negative refraction has been experimentally illustrated in such a MIM waveguide^23^, direct demonstration of backward SPP wave in a plasmonic waveguide with clear anti-parallel phase and group velocities is still lacking. This may be attributed to the strong damping nature of SPPs which leads to a propagation length of only few wavelengths in these plasmonic waveguides^20^,^22^.

The SPPs cannot be directly scaled to lower frequencies because metal fundamentally behaves as perfect electric conductor below its plasma frequency at far infrared. In order to make use of the high field confinement and enhancement in subwavelength scale of the SPP wave, plasmonic metamaterials consisting of metal surface textured with subwavelength-scale corrugations or dimples have been proposed whose surface plasmon frequency can be tailored by the geometrical parameters of the structures^24^,^25^,^26^,^27^,^28^,^29^,^30^,^31^,^32^,^33^. These so-called “spoof” or “designer” SPPs possess the similar dispersion relations and field properties of optical SPPs, but work at lower frequency down to microwave or terahertz regimes. Recently, a more practical structure — the ultrathin corrugated metallic strip, has been proposed to propagate conformal surface plasmons (CSPs) on arbitrarily curved surfaces^34^, which is proved to be a potential candidate for SPP device and circuit applications in microwave and terahertz frequencies^35^,^36^,^37^,^38^.

The spoof SPPs should support backward wave as they inherit most of the exotic features of their optical counterpart. A recent theoretical study shows that deep-subwavelength negative-index waveguiding can be obtained in two closely coupled CSP waveguides mimicking an MIM optical system^39^. However, to the best of our knowledge, clear experimental demonstration of backward propagation of spoof surface plasmonic wave with opposite directions of phase and group velocities has not yet been reported. In this work, we propose a new design of plasmonic metamaterial to construct a symmetric CSP waveguide mimicking an IMI system, which can support backward wave propagation with its odd guiding mode. Thanks to the low loss and large propagation length of the new spoof SPPs waveguide, we achieve the dispersion relation of the odd mode with a negative slope from both the full wave simulation and direct measurement of the transmission through the waveguide. Such results insure anti-parallel phase and group velocities, and the backward wave propagation can be clearly verified by the phase evolution in the simulation. To further apply the backward wave propagation, we also design a contra-directional coupler in the microwave band that can route the input signal to opposite directions with forward or backward coupling at different frequencies. Experimental test on the fabricated prototype coupler have validated the design principle based on the backward wave propagation in the plasmonic metamaterial waveguide. We believe these results could contribute to the development of more complicated surface circuitry for microwave and terahertz wave.

## Results

### Dispersion analysis and backward wave propagation

The traditional CSP waveguide is composed of an ultra-thin metallic strip with corrugated periodic grooves on one side that could support well confined surface wave propagation^34^. It imitates the optical SPPs waveguiding at the interface between the semi-infinite dielectric and metal media. However, to support backward propagation, symmetric CSP or close coupled CSP waveguides should be considered which mimics an IMI or a MIM tri-layer planar waveguide at optical range, respectively. In such tri-layer planar waveguide, the guided-mode dispersion relation is decomposed into two branches owing to the interaction of SPPs at the upper and lower interfaces, and the higher-frequency branch (anti-symmetric mode, or odd mode) could appears a negative slope indicating backward wave^18^,^19^,^20^,^21^,^22^.

**Figure 1:**
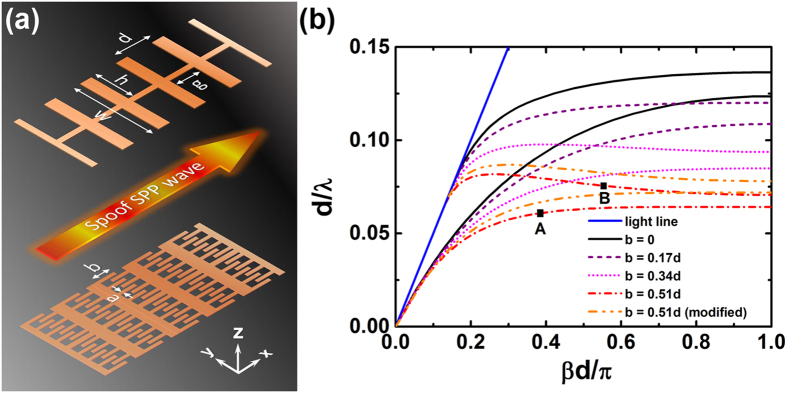
Structure and dispersion relations of the CSP structures. (**a**) Geometry of the traditional (top) and the proposed (bottom) symmetric CSP structures. (**b**) Dispersion curves for the different symmetric CSP structures. The modified one (orange dashed line) indicates the dispersion curve for the CSP pattern in which four small grooves inside each slot near the centric strip have been removed. Only the fundamental modes have been plotted. The metallic strip is considered as PEC with negligible thickness and the geometric parameters are set as *g* = 0.67*d*, *h* = 1.6*d*, *a* = 0.1*d*, *w* = 3.4*d*, respectively.

Here we focus on the plasmonic metamaterial of symmetric CSP structure which has same corrugated grooves on both sides of the strip, as showed in top part of Fig. 1a. The guide-mode dispersion relations are calculated numerically and displayed in Fig. 1b. The dispersion curves split into a lower- and a higher-frequency band, corresponding to the bonding and antibonding combination of the spoof SPP waves at the both sides of the strip. However, either the even mode (lower-frequency branch) or the odd mode (higher-frequency branch) has a positive slope whatever the geometric parameters (the gap width or the height) of the grooves are chosen.

**Figure 2:**
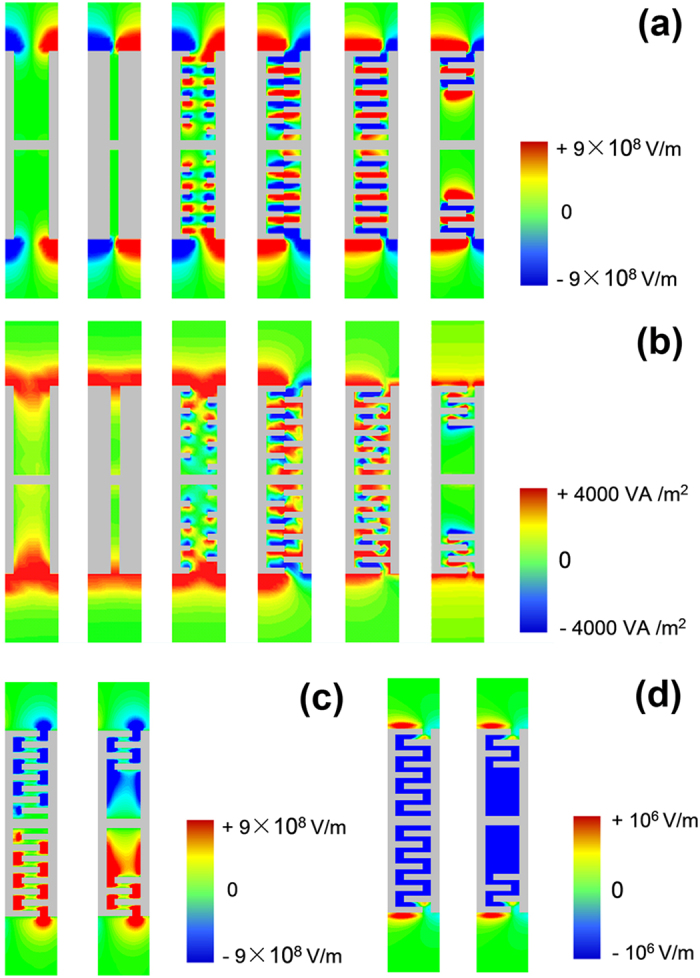
Simulated EM field and energy flux distribution in a unit-cell of the symmetric CSP waveguide (at *β*d/π = 0.5). The distributions of (**a**) the *y* component of electric field, (**b**) the EM energy flux along *x* direction, (**c**) the *x* component of electric field, and (**d**) the *z* component of magnetic field for different slot structures. The parameters of the slot in (**a**) or (**b**) are set from left to right as *g* = 0.67*d*, and *b* = 0; *g* = 0.33*d*, and *b* = 0; *g* = 0.67*d*, and *b* = 0.17*d*; *g* = 0.67*d*, and *b* = 0.34*d*; *g* = 0.67*d* and *b* = 0.51*d*; *g* = 0.67*d* and *b* = 0.51*d* with simplified grooves; while other parameters are the same as that in Fig. 1b. EM power is fed from the left to the right.

For the optical counterpart, the IMI tri-layer planar waveguide, it has been found that in order to obtain a backward wave, the power flow inside the metal layer must be greater (in absolute value) than that in the outside insulator media^22^. We simulate the EM field distribution for the odd mode in the symmetric CSP strip waveguide and find that the EM power is merely confined at the two edges of the CSP strip as shown in Fig. 2a,b. The EM power can hardly penetrate into the slots in the metallic strip leading to an always forward wave propagation in the CSP waveguide. In order to couple the EM wave into the slots we modify the traditional symmetric CSP by adding small metallic grooves at both sides of the slots as shown in the bottom part of Fig. 1a. The resulted interdigital structure inside each slot further enhances the capacitive coupling of the EM fields from both sides of the strip and the power flow could then penetrate into the slot region. This is clearly illustrated in the field and Poynting vector distributions shown in Fig. 2a,b. As increasing the length (*b*) of the small grooves of the interdigital structure, the EM field is more and more enhanced inside the slots and the power flow is more and more concentrated in the strip region. The power flow inside the strip region may become greater than that in the two edge regions. As a result, the dispersion relations of the modified CSP waveguide structure move to lower frequency, and after a certain critical point the dispersion curve of the odd mode begins to present negative slope at large *β*. This means the odd guiding mode will support backward wave with anti-parallel phase and group velocities. The anti-symmetric transverse electric field (*E*_x_) and the only magnetic field (*H*_z_) are also plotted in Fig. 2c,d respectively, which accord with the field pattern of an odd TM spoof SPPs mode. It is noted that the dispersion curve of the spoof SPPs for the higher-frequency branch does not start at the origin, as only non-radiative modes are taken into consideration.

For practical consideration we further modify the CSP pattern by removing four small grooves inside each slot near the centric strip. The simplified structure does not change its dispersion characteristic much, but will experiences less propagation loss. We have added the dispersion curve of the simplified CSP waveguide in comparison with that of the ideal patterns in Fig. 1b. The EM field and the energy flux distributions for the simplified CSP waveguide is also compared in Fig. 2. It shows that the simplified CSP pattern has similar dispersion curve supporting backward wave as well as similar field patterns compared with that of the ideal CSP pattern.

**Figure 3:**
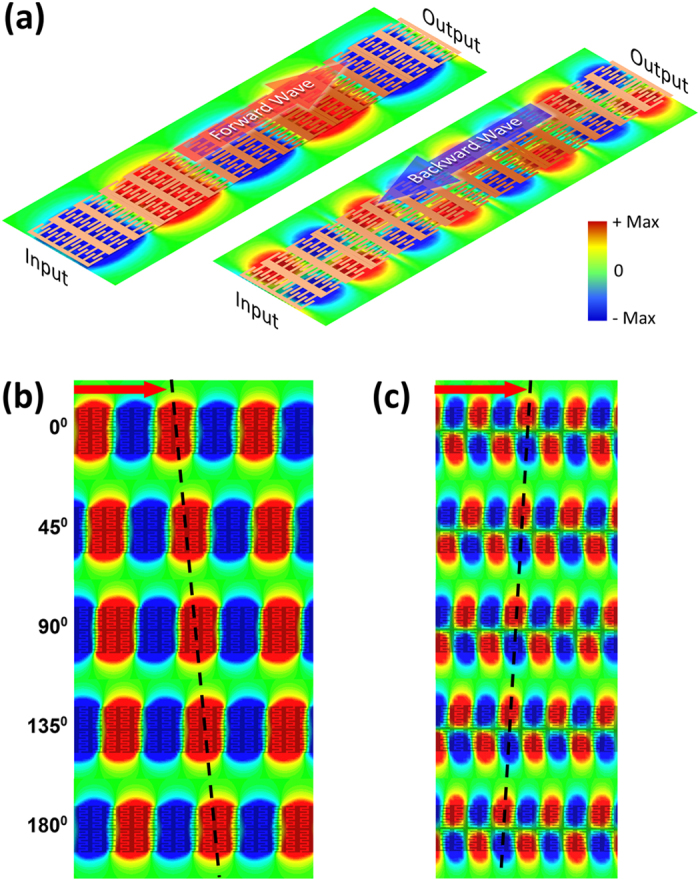
Transverse electric field (*E*_z_) distribution (**a**) and its evolution ((**b**, **c**)) along the proposed CSP structure. Left or right parts of (**a**) corresponds to point A or B as marked in Fig. 1b indicating a forward or a backward wave propagation, respectively. (**b**) and (**c**) demonstrate the field evolution of the forward and backward wave corresponding to point A and B as marked in Fig.1b. The black dashed line denotes the phase front in the field evolution, and the red arrow in (**b**) and (**c**) indicates the direction of EM power flow.

To clearly demonstrate the backward propagation in the proposed symmetric CSP waveguide, we calculate the EM wave evolution along an ideal long waveguide. Figure 3a demonstrates the transverse electric field distribution close to the surface of the metallic strip. Symmetric and anti-symmetric transverse field patterns have been obtained corresponding to the even (left of Fig. 3a) and the odd guiding modes (right of Fig. 3a) respectively, and both have tightly confined field concentrated in the metallic strip region illustrating typical features of spoof SPPs modes with little scattering loss. In Fig. 3b,c, snap shots of the field patterns travelling along the CSP waveguide are demonstrated at different moments. The group velocity is determined by the EM power propagation which is fed from the left end and flows to the right end in Fig. 3b,c. The wave front indicated by the black dashed line clearly exhibits that the electric field evolves to the right for the lower-frequency even mode, but inversely to the left for the higher-frequency odd mode. This unique feature indicates that the even mode supports a forward wave, while the odd mode supports a backward wave with anti-parallel phase and group velocities. The Supplementary movie S1 or S2 for the wave propagation of the even or odd guiding mode can be found in the Supplementary Informations.

### Verification by transmission measurement

**Figure 4:**
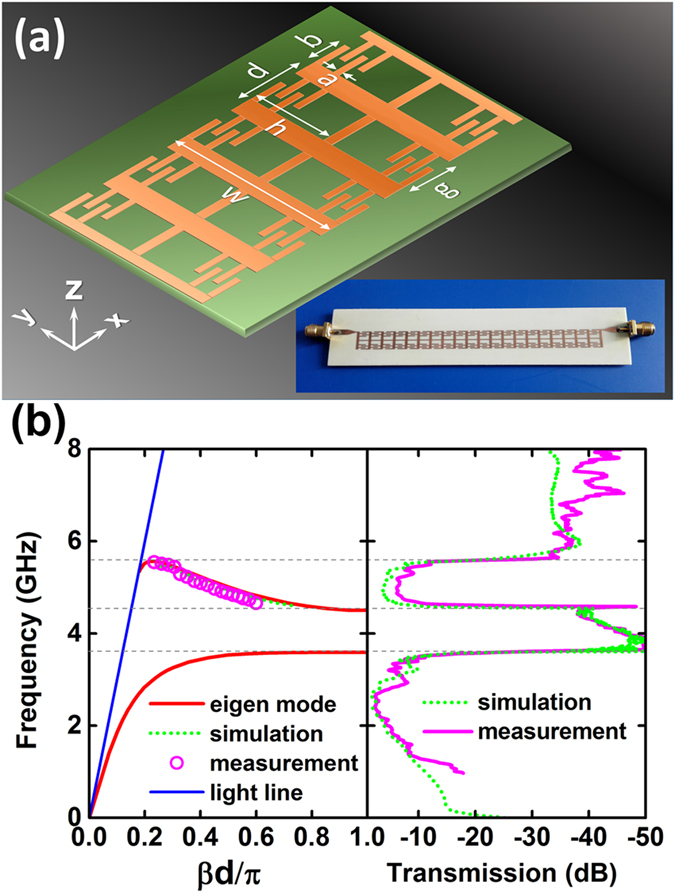
Structure and properties of the proposed practical symmetric CSP waveguide. (**a**) The schematic of the waveguide and the photo of the fabricated prototype. (**b**) Calculated and measured dispersion curves and the transmission spectrum of the CSP waveguide. The 0.018 mm thick copper strip with geometric parameters of *d* = 5 mm, *g* = 4 mm, *h* = 3.6 mm, *w* = 7.5 mm, *a* = 0.3 mm, and *b* = 3 mm is printed on the dielectric substrate.

To verify the backward wave propagation in the proposed plasmonic metamaterial structure, we design a practical symmetric CSP waveguide based on the previous analysis. The waveguide is composed of ultrathin copper strip with previously proposed structure on top of a dielectric substrate designed to work at microwave range. Simplified pattern is used by removing four small grooves inside each slot near the centric strip as shown in Fig. 4a. Figure 4b shows the calculated dispersion relation (red line) through the eigen-mode analysis, which demonstrates an even mode with normal dispersion and an odd mode with dispersion curve possessing negative slope.

To test the propagation characteristics, several prototype waveguides have been fabricated and the measured transmission spectrum is presented and compared with the full-wave simulations in Fig. 4b. Two passbands appear in the transmission spectrum with good agreement between simulation and measurement, which corresponds to the even and odd guiding modes. Although the transmission is slightly low (around -3 dB in simulation and -5 dB in measurement for the second passband) which can be attributed to the metallic and dielectric loss and the un-optimized impedance match at the input and output ends, the two passbands exactly agree with the calculated dispersion curves. We can also extract the wavenumber *β* from transmission measurements of the CSP waveguides with different length by de-embedding the contributions from the connectors and input and output transitions. The extracted data for the second passband corresponding to the odd guiding mode is displayed in Fig. 4a (the open circles), which is in accordance with either the calculation from the eigen mode analysis or the full wave simulation. The extracted dispersion curve clearly indicates a negative slope in the whole passband, therefore provides a verification of the backward wave propagation in this practical plasmonic metamaterial structure.

### Application of backward wave: contra-directional coupler

Backward EM wave propagation is the foundation of many extraordinary phenomena, such as the negative refraction, or the perfect lens with sub-diffraction resolution. The backward wave in left-handed transmission line has also been utilized in designing novel microwave components and antennas. As a further validation and a direct application of the backward spoof SPP wave, we present the design and performance of a contra-directional EM wave coupler.

**Figure 5:**
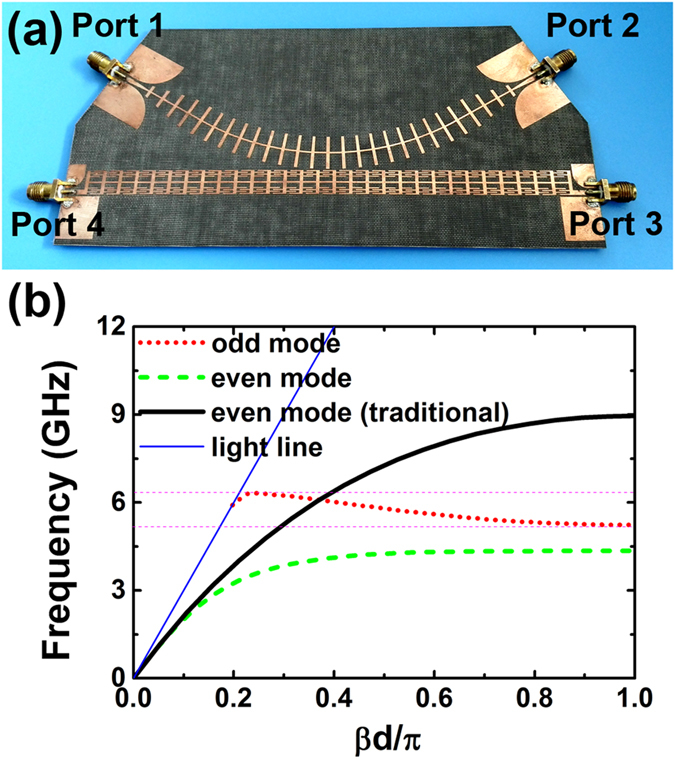
The coupler and the corresponding dispersion curves. (**a**) The photograph of the fabricated prototype coupler with SMA connectors mounted at the four terminals. (**b**) Simulated dispersion relations for both the traditional and the modified CSP waveguides. The geometric parameters are set as *d* = 5 mm, *g* = 4 mm, *h* = 5 mm, *w* = 10.5 mm for the traditional CSP structure, while *d* = 5 mm, *g* = 4 mm, *h* = 3.5 mm, w = 7.5 mm, *a* = 0.3 mm, *b* = 3 mm for the modified CSP structure, respectively. The gap between the two waveguides is optimized at 0.6 mm.

The composite right/left-handed microstrip line supporting the backward wave was successfully applied in designing backward directional coupler at microwave frequency^40^,^41^. Similar idea was also extended to the optical domain in a theoretical work on a contra-directional coupler through stacked IMI SPP waveguides^42^. Here we utilize the previously proposed plasmonic metamaterial structure to construct the contra-directional coupler in the microwave frequency band. The proposed coupler is composed of a straight symmetric CSP strip with the modified slots and a bending traditional symmetric CSP strip with ordinary straight slots. The two strip waveguides are placed together with a small coupling gap at the middle forming a four port microwave device, as shown in Fig. 5a. We first analyze the guiding spoof SPP modes in the two CSP waveguides for a specific design with the parameters listed in the figure caption. The calculated dispersion relations are displayed in Fig. 5b. The traditional CSP strip can only support the fundamental even mode below 9 GHz, while the modified CSP strip supports the even mode below 4.5 GHz but the odd mode from 5.2 to 6.3 GHz. The odd mode is obviously a backward wave as it has a negative-sloped dispersion curve.

**Figure 6:**
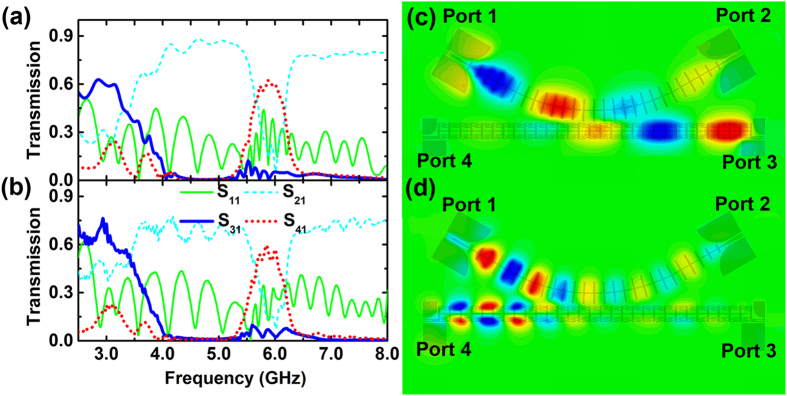
Transmission spectrum and electric field distributions. The simulated (**a**) and the measured (**b**) transmission spectrum of the coupler. The simulated transverse electric field (*E*_Z_) distributions along the device at 2.8 GHz for the forward coupling (**c**), and at 6.03 GHz for the backward coupling (**d**).

To study the wave coupling behavior we have fabricated a prototype device as shown in Fig. 5a. The signal is fed from left end (Port 1) of the traditional CSP waveguide and the coupling behavior is studied by measuring the *S* parameters between input and the other three output ports. According to the coupled mode theory, the coupling between two adjacent waveguides is determined by the propagation constants of the two waveguides^43^. When the two waveguides have same or similar values of propagation constant, the EM energy propagating in one waveguide may couple to the other and the coupling strength also depends on the gap and the coupling length of the two waveguides. As indicated in Fig. 5b, the wave propagation is dominated by the even mode in the traditional CSP waveguide, and it can be coupled to the even mode of the modified CSP waveguide at frequency below 3.5 GHz, as the dispersion curves of the two modes are close to each other. Both even modes in traditional and the modified CSP strips support the forward wave, therefore a large among of energy may be coupled from the traditional CSP to the modified CSP waveguide resulting in an output to the Port 3. It is noticed that the dispersion curve of the even mode in the traditional CSP waveguide will cross that of the odd mode in the modified CSP waveguide leading to a coupling between these two guiding modes at around 6 GHz. As the odd mode in the modified CSP waveguide supports the backward wave, we can expect that the EM energy coupled from the traditional CSP waveguide will propagate backwardly and result in an output to the Port 4. These properties have been verified by both the simulated and the measured *S* parameters which are displayed in Fig. 6a,b. Clear EM energy coupling between the traditional and the modified CSP waveguides is observed either in the frequency band below 3.5 GHz, or in the frequency band around 6 GHz. The coupling at lower band is caused by the forward coupling between the even modes in both waveguides and produces a large transmission coefficient from Port 1 to Port 3 (*S*_31_). While the coupling around 6 GHz is attributed to the backward coupling between the even mode in the traditional CSP waveguide and the odd mode in the modified waveguide and on the contrary produces an obvious transmission coefficient from Port 1 to Port 4 (*S*_41_). The simulation and the measurement agree with each other well. We also notice that the coupling strength can be changed by altering the length of the coupling section or the gap between the two strips due to the spatial field variation along the waveguides.

To visualize the forward and backward coupling in the two bands, we illustrate the different transverse electric field distributions in Fig. 6c,d. When operated in the lower band, the EM wave propagating in the traditional CSP waveguide couples with most of the energy to the nearby modified CSP waveguide in the middle part and continues to propagate to Port 3. However, at the higher band the coupled wave in the modified CSP waveguide supports only the odd mode of backward wave with Poynting vector anti-parallel to the wave-vector, thus propagates contra-directionally to the Port 4. The demonstrated coupler utilizes both the even and odd spoof SPP guiding modes that support forward and backward wave, and can route the microwave signal to opposite terminals at different operating frequencies. This unique property may offer for instance alternative designs of components and devices in the communication systems.

## Discussion

In summary, we have presented clear demonstration of backward spoof SPP wave propagation in a specially designed plasmonic metamaterial. To mimic the optical IMI tri-layer structure that has been theoretically predicted to support backward guiding mode, we modified a symmetric corrugated metallic strip by integrating interdigital structure in each slot to further enhance and concentrate EM field in the strip. The resulted CSP waveguide could have odd guiding mode with dispersion curve possessing negative slope. We have demonstrated that such odd mode could support backward propagation from both the simulated EM field evolution and the transmission measurement of fabricated prototype plasmonic metamaterial structure. We remark that the demonstration of the backward wave propagating in the plasmonic metamaterial of single metallic structure with strong field confinement and low scattering loss has not been reported, to the best of our knowledge. As a further validation and a direct application of the backward wave propagation, a microwave contra-directional coupler has been designed and tested that could route the microwave signal to opposite ends through forward or backward coupling at different operating frequencies. Backward wave is the fundamental of many peculiar EM phenomena such as the negative refraction or the sub-diffraction imaging, therefore our findings can lead to many exciting applications based on the backward surface wave propagation in plasmonic metamaterial. We also believe that the proposed planar plasmonic structure can be easily extended to higher frequency regime and may be very promising for further development of practical ultrathin surface circuitry for microwave and terahertz radiation.

## Methods

### Simulation

The eigen mode analysis, the calculation of the transmission spectrum as well as the EM field distribution of the proposed spoof SPP waveguide are carried out with the help of commercial software, the CST Microwave Studio. In the simulation the substrate used in Fig. 4, has a permittivity of 3.55, a loss tangent of 0.0027 and a thickness of 0.813 mm, while the substrate used in Fig. 5, has a permittivity of 2.55, a loss tangent of 0.0035 and a thickness of 0.5 mm. All the metal films are considered as copper with a conductivity of 5.8 × 10^7^ S/m.

### Sample fabrication

The prototypes of the CSP waveguide and the contra-directional coupler are made by the commercial print circuit broadband (PCB) technique.

### Measurement

To test the propagation characteristics, several prototype waveguides have been fabricated with long strip including different numbers of unit cells. Two standard SMA connectors are welded to the ends of the strip (offset to the edge to stimulate anti-symmetric odd mode more easily) to couple in and out the microwave power, as shown in the inset of Fig. 4a. The fabricated contra-directional coupler is shown in Fig. 5a. In order to improve impedance match, a transition section of co-planar waveguide (CPW) with a flaring ground and gradient center conductor width is employed to connect between the SMA connector and the CSP strip at the four ports. It is noted that the CPW is connected to the edge of the modified CSP strip so as to improve the impedance match for the anti-symmetric odd mode. Both the transmission spectrum of the waveguide and the *S* parameters of the coupler device have been measured through an Agilent Vector Network Analyzer (N5244A).

## Additional Information

**How to cite this article**: Liu, X. *et al.* Backward spoof surface wave in plasmonic metamaterial of ultrathin metallic structure. *Sci. Rep.* **6**, 20448; doi: 10.1038/srep20448 (2016).

## Supplementary Material

Supplementary Information

Supplementary Movie 1

Supplementary Movie 2
