# Supplementary material for: Backward spoof surface wave in plasmonic metamaterial of ultrathin metallic structure
Source: Sci Rep. 2016 Feb 4;6:20448. doi: 10.1038/srep20448 (PMC4740866; doi:10.1038/srep20448)
Supplement: Supplementary Information [file srep20448-s1.pdf]

Supplementary Information

Backward spoof surface wave in plasmonic metamaterial of ultrathin metallic structure

Xiaoyong Liu, Yijun Feng, * *Bo Zhu, Junming Zhao, Tian Jiang*

Department of Electronic Engineering, School of Electronic Science and Engineering, Nanjing University, Nanjing, 210093, China

Supplementary movie S1: Field1.mov (Transverse electric field evolution of even mode along the proposed CSP waveguide corresponding to point A as marked in Fig. 1b.)

Supplementary movie S2: Field2.mov (Transverse electric field evolution of odd mode along the proposed CSP waveguide corresponding to point B as marked in Fig. 1b.)
